# Bridging the Gap in Secondary Antibody Deficiencies: Current Evidence and Unmet Needs in Diagnosis and Management with Immunoglobulin Replacement

**DOI:** 10.1007/s12016-025-09116-4

**Published:** 2025-12-06

**Authors:** Andy Ka Chun Kan, Ben Chun Yin Chan, Chak Sing Lau, Philip H Li

**Affiliations:** Division of Rheumatology and Clinical Immunology, Department of Medicine, Queen Mary Hospital, The University of Hong Kong, Hong Kong, Hong Kong

**Keywords:** Secondary antibody deficiency, Immunodeficiency, Epidemiology, Therapeutics, Subcutaneous immunoglobulin replacement, Infection

## Abstract

Secondary antibody deficiency (SAD) represents a substantial yet under-recognised global healthcare burden. It is more prevalent than primary antibody deficiency, but frequently under-diagnosed and variably managed worldwide. Prompt diagnosis is often hindered by insufficient awareness among clinicians, lack of global consensus on screening/monitoring for SAD among at-risk patients, inadequate clinical immunology services and lack of standardised referral pathways/protocols. Management practices vary widely, with little international agreement, particularly regarding threshold to initiate immunoglobulin replacement, as well as regimen, dosage and frequency of immunoglobulin administration. Subcutaneous immunoglobulin (SCIg) replacement emerged as a promising alternative to traditional intravenous immunoglobulin (IVIg) replacement. IVIg requires monthly infusions in inpatient/day-hospital settings leading to high peak serum IgG and subsequent variations with end-of-cycle ‘wear-off effect’, causing more systemic side effects and increased risk of breakthrough infections, and disruption of daily life and employment. While previous evidence was largely derived from primary antibody deficiency, recent comparative studies on SAD patients indicate that SCIg replacement, through weekly self-administered infusions, can achieve more stable and higher trough serum IgG, lower infection rates, fewer systemic adverse reactions and enhanced health-related quality-of-life compared to IVIg. There is also potential cost-savings from the use of SCIg replacement. This review emphasises the urgent need for standardised guidelines on screening/diagnosis and treatment of SAD, and large-scale multi-centre trials and real-world studies on IVIg vs SCIg replacement among SAD patients, which will facilitate better identification, management, and health-outcomes for SAD patients, ultimately alleviating a significant global health challenge through coordinated clinical, research, and policy efforts.

## Introduction

Immunodeficiency refers to defects in the immune system that lead to an increased susceptibility to infections, autoimmune or autoinflammatory diseases, and/or malignancies. Immunodeficiencies are categorised based on the component of the immune system affected, with antibody deficiencies accounting for around half of all cases [1–3]. Antibody deficiencies can also be classified by aetiology into primary (inborn errors of immunity) or secondary (consequences of other diseases or treatment) [4]. Secondary antibody deficiency (SAD) can be due to impairment in antibody production (e.g. haematological malignancies, cytotoxic/immunosuppressive treatment) or antibody loss (e.g. nephrotic syndrome, protein-losing enteropathy) [5]. Patients with SAD due to antibody loss have reduced quantity of antibodies but intact specific antibody production, and hence may have a better prognosis compared to those with SAD due to impairment in antibody production [6]. 

SAD represents a huge but under-recognised global healthcare burden [6, 7]. Due to limited available data, its current management is largely extrapolated from treatment principles established for primary antibody deficiency, despite important clinical distinctions between the two entities [8, 9]. Furthermore, there is a lack of global consensus on the definition or treatment thresholds for SAD, contributing to significant variability in clinical management across regions and institutions. Treatment strategies for antibody deficiencies generally include antibiotic prophylaxis, vaccination, and immunoglobulin replacement therapy. Intravenous immunoglobulin (IVIg) is typically administered as a monthly infusion in a day-hospital setting, requiring several hours per session. In recent years, subcutaneous immunoglobulin (SCIg), which can be self-administered at home on a weekly basis, has become increasingly popular. However, most clinical studies to date have focused on its use in patients with primary antibody deficiency, leaving a gap in evidence-based guidance for those with SAD.

In this review, the global epidemiology and disparities in the management of SAD will be discussed. Furthermore, the efficacy, real-world outcomes and cost-effectiveness of treatment for SAD, including SCIg, will also be examined.

References for this review article were identified by searches of PubMed using the search terms ‘secondary antibody deficiency’, ‘immunodeficiency’, ‘prevalence’, ‘guideline’, ‘immunoglobulin replacement’, ‘IVIg’, ‘SCIg’, ‘IgG’, ‘infection outcomes’, ‘adverse event’ and ‘quality of life’, as well as from the authors’ personal collection of literature. The final list of references was determined on the basis of relevance to the focus of this review article.

## Global Epidemiology, Underdiagnosis and Disparities in Management of SAD

### Global Epidemiology of SAD

SAD is significantly more prevalent than previously recognised. While primary antibody deficiency affects approximately 1 in 25,000 individuals worldwide (around 320,000 people), SAD is estimated to be up to 30 times more common, underscoring its substantial yet under-appreciated public health burden [5, 10]. A study conducted at an adult tertiary/quaternary referral centre in Hong Kong found that SAD accounted for the majority of all patients receiving immunoglobulin replacement, while primary antibody deficiency accounted for less than 5% [1]. The estimated prevalence of SAD of various aetiologies is summarised in Table 1.

**Table 1:** Prevalence of common causes of secondary antibody deficiency

Variables	Prevalence of the disease	Prevalence of secondary antibody deficiency	Estimated population with secondary antibody deficiency †
*Haematological malignancies*			
Chronic lymphocytic leukaemia	1 in 14,000[11–13]	58.7%[14]	343,814
Multiple myeloma	1 in 20,000[13, 15]	80–90%[16–18]	328,000–369,000
Non-Hodgkin lymphoma	1 in 3,400[13]	14% (24% in DLBCL)[19, 20]	337,647
*Protein-losing states*			
Lupus nephritis (proteinuria)	1 in 4,700[21]	16% (30% among those with nephrotic range proteinuria)[22]	279,149
Protein-losing enteropathy	Rare	72%[23]	–
*Iatrogenic*			
Rituximab	–	17–56%[24–27]	–
Corticosteroids (for giant cell arteritis/polymyalgia rheumatica)	–	58%[28]	–
Mycophenolate mofetil	–	39%[29]	–
Cyclophosphamide	–	30%[22]	–
*Transplantation*			
Haematopoietic stem cell transplantation		53%[30]	–
Solid organ transplantation	–	45%[31]	–
Lung transplantation	–	63%[31]	–
Heart transplant	–	49%[31]	–
Kidney transplantation	–	40%[31]	–
Liver transplantation	–	16%[31]	–

The table only included causes of secondary antibody deficiency with prevalence data available in the literature

† Based on the estimated world population of 8.2 billion in 2024, according to the United Nations

*DLBCL* diffuse large B-cell lymphoma

### Under-Diagnosis and Diagnostic Challenges of SAD

SAD patients may remain asymptomatic despite significant hypogammaglobulinaemia or present with apparently normal total immunoglobulin levels in patients with immunoglobulin subclass or specific antibody deficiencies. Nonetheless, they can already be at risk of serious or life-threatening infections [6]. Prompt diagnosis through screening immunoglobulin levels of at-risk patients (e.g. haematological malignancy, use of immunosuppressants or B-cell depleting therapies), and timely initiation of treatment could reduce the risk of serious or life-threatening infections in these patients [32, 33]. Serial monitoring of immunoglobulin levels (IgG, IgA and IgM) is usually sufficient for screening and monitoring for SAD; with additional immunological investigations, such as specific antibodies/vaccine responses and IgG subclass (especially when total IgG levels are normal), lymphocyte subset, B-cell phenotyping and lymphocyte proliferation test selectively performed based on relevant clinical context, and especially at baseline [34]. However, screening and monitoring for SAD among at-risk patients have yet to be common practices among all clinicians, and patients have often been diagnosed only after they became symptomatic or suffered from significant infections. Among those who received a formal diagnosis of SAD, significant diagnostic delay has been observed in multiple real-world cohort studies, with a median of around 1 year [35, 36]. 

Several factors may contribute to the under-diagnosis of SAD. Firstly, at-risk patients are chiefly managed by non-immunologists, many of whom have limited awareness or familiarity with SAD [35, 37, 38]. This can lead to missed or delayed recognition of the condition, particularly in complex clinical settings where immune dysfunction may be overshadowed by other comorbidities or treatments. There could be insufficient understanding of the polyclonal nature of IgG among non-immunologists. Although hypogammaglobulinaemia (low serum IgG levels) is the most common manifestation of antibody deficiency, a normal serum IgG level does not exclude antibody deficiency, as functional specific antibodies against a variety of pathogens are required to confer protection against various infections. For example, patients with immunoparesis could suffer from significant antibody deficiency despite a normal serum IgG level, as the majority of circulating IgG are non-functional [39]. Insufficient understanding of this concept among non-immunologists could contribute to the failures to diagnose, inadequate diagnostic evaluations, diagnostic delays, inappropriate treatment delays, and inadequate immunoglobulin replacement regimens. Secondly, there is a lack of global consensus on screening and monitoring for SAD, as well as disparity or lack of incorporation into disease-specific guidelines, as shown in Table 2. For example, while the American Academy of Allergy, Asthma & Immunology (AAAAI) guideline recommended checking immunoglobulins at diagnosis and every 6 months for chronic lymphocytic leukaemia [32], a number of guidelines for chronic lymphocytic leukaemia only recommended checking immunoglobulin profile before treatment without specific and clear recommendations on subsequent monitoring, or even did not provid any guidance on screening or monitoring for SAD at all (Table 2) [40, 43]. Thirdly, scarcity of clinical immunology services, as well as the lack of established referral pathways/protocols for suspected/confirmed SAD patients, may hinder prompt diagnosis and treatment [54]. Disparity in the availability of immunology service exists among different countries. Many countries in Europe, including Germany (6.04 per 100,000 population), Hungary (5.14 per 100,000 population), France (2.00 per 100,000 population) and Spain (3.24 per 100,000) have a ratio above 1 immunology and allergy specialist per 100,000 population. In the United States, there has been a decline in the number of immunologists per population over the past 2 decades, but the ratio is still relatively high at 1.08 immunology and allergy specialists per 100,000 population in 2020 [55]. On the contrary, most countries in Asia-Pacific have a substantially lower ratio, with Australia (0.71 per 100,000 population), New Zealand (0.23 per 100,000 population), Hong Kong (0.09 per 100,000 population), Thailand (0.1 per 100,000 population) and Malaysia (0.0004 per 100,000 population) having a ratio below 1 per 100,000 population, except for Japan (1.63 per 100,000) and the Philippines (1.51 per 100,000 population) [56, 57].

**Table 2:** Summary of the disparities in disease/aetiology-specific guidelines on the screening and monitoring, as well as treatment of secondary antibody deficiency

Disease/aetiology	Society	Year	Recommendations on screening and monitoring	Recommendations on treatment
*Haematological malignancies*				
Chronic lymphocytic leukaemia	ESMO[40]	2021	Check immunoglobulin profile before treatment, at the end of therapy, and during follow-up	Use of antibiotic and antiviral prophylaxis in those with recurrent infections and/or very high risk of developing infections. Immunoglobulin replacement only in those with severe hypogammaglobulinaemia and repeated/severe infections.
	BSH[41]	2022	No specific recommendations on screening or monitoring.	Immunoglobulin replacement shall be offered for those with serum IgG < 400 mg/dL, recurrent or serious infection despite antibiotic prophylaxis for 6 months and a documented vaccine response failure.
	NCCN[42]	2024	No specific recommendations on screening or monitoring.	Immunoglobulin replacement in those with serum IgG < 500 mg/dL with recurrent sinopulmonary infections requiring intravenous antibiotics or hospitalisation.
	iwCLL[43]	2018	Check immunoglobulin profile before treatment. No specific recommendations on subsequent monitoring for antibody deficiency	IVIg should be reserved to individual situations of hypogammaglobulinaemia and repeated infections.
Multiple myeloma	NICE[44]	2016	Smouldering myeloma: check immunoglobulin profile every 3 months for the first 5 years, then decide the frequency based on the long‑term stability of the disease. Completed myeloma treatment and recovered: check immunoglobulin profile at least every 3 months.	Consider IVIg therapy for those with hypogammaglobulinaemia and recurrent infections.
*Iatrogenic*				
Rituximab (rheumatoid arthritis)	BSR and BHPR[45, 46]	2011, 2018	Check immunoglobulin profiles before initiating treatment, 4 to 6 months after infusions, and before any re-treatment.	No specific recommendations on treatment.
Immunosuppressants (systemic lupus erythematosus)	BSR[47]	2018	Check immunoglobulin profiles before initiating drugs at risk of inducing immunodeficiency (e.g. rituximab, mycophenolate mofetil, cyclophosphamide), then 3 to 6 months later, then annually.	No specific recommendations on treatment.
Rituximab (rheumatoid arthritis)	EULAR[48]	2022	No specific recommendations on screening or monitoring.	No specific recommendations on treatment.
Rituximab (rheumatoid arthritis)	ACR[49]	2021	No specific recommendations on screening or monitoring.	No specific recommendations on treatment.
Rituximab (ANCA-associated vasculitis)	EULAR[50]	2024	Check immunoglobulin profile before each course of rituximab.	No specific recommendations on treatment.
Rituximab (ANCA-associated vasculitis)	ACR and VF[51]	2021	No specific recommendations on screening or monitoring.	Immunoglobulin replacement in those with serum IgG < 300 mg/dL and recurrent severe infections, or without recurrent severe infections but having impaired immune responses to vaccines.
*Transplantation*				
Haematopoietic stem cell transplantation	ASBMT and CBMTG[52]	2018	No specific recommendations on screening or monitoring.	Immunoglobulin replacement can be considered in umbilical cord blood transplant recipients, paediatric patients undergoing transplantation for inherited or acquired disorders associated with B-cell deficiency, and chronic GVHD patients with recurrent sinopulmonary infections.
	ASBMT[53]	2009	No specific recommendations on screening or monitoring.	Immunoglobulin replacement is considered in patients with ‘severe’ hypogammaglobulinaemia (< 400 mg/dL).

*ACR* American College of Rheumatology, *ANCA* anti-neutrophil cytoplasmic antibody, *ASBMT* American Society for Blood and Marrow Transplantation, *BHPR* British Health Professionals in Rheumatology, *BSH* British Society for Haematology, *BSR* British Society for Rheumatology, *CBMTG* Canadian Blood and Marrow Transplant Group, *ESMO* European Society for Medical Oncology, *EULAR* European Alliance of Associations for Rheumatology, *GVHD* graft-versus-host disease, *iwCLL* International Workshop on Chronic Lymphocytic Leukemia, *NCCN* National Comprehensive Cancer Network, *NICE* National Institute for Health and Care Excellence, *VF* Vasculitis Foundation

### Disparities in the Management of SAD

The management of SAD remains a challenging and sometimes ambiguous area of clinical immunology, largely due to the lack of high-level evidence on treatment efficacy, and hence the lack of globally accepted guidelines and the reliance on expert opinion or small studies [5, 32, 58–60]. This has resulted in substantial variability in clinical practice, with disparities particularly evident in three key areas: (1) threshold to start immunoglobulin replacement, (2) regimen, dosage and frequency of immunoglobulin replacement, and (3) conditions to stop immunoglobulin replacement.

Hypogammaglobulinaemia is defined as a low serum IgG level (below 2 standard deviations for age, ranging from less than 500 to 700 mg/dL depending on the assay methodology), with moderate and severe hypogammaglobulinaemia usually referring to a serum IgG level less than 400 mg/dL and 200 mg/dL respectively [32, 61]. Treatment for SAD is generally indicated when there is a history of significant infection(s) with evidence of antibody deficiency, either in quantity (e.g. hypogammaglobulinaemia, immunoglobulin subclass deficiency) or quality (e.g. specific antibody deficiency, impaired vaccine response), or moderate/severe hypogammaglobulinaemia regardless of history of infections [32, 61]. However, there is no consensus on the threshold to start treatment, and recommendations vary across different disease-specific guidelines, summarised in Table 2. Hence, the AAAAI has made a step forward to propose a universal framework, in which immunoglobulin replacement is indicated in any SAD patients with history of severe/unusual/recurrent infections and serum IgG < 700 mg/dL plus reduced IgA/IgM and impaired vaccine response, history of severe/unusual/recurrent infections and serum IgG < 400 mg/dL, history of severe/unusual/recurrent infections and failing antibiotic prophylaxis, or serum IgG < 150 mg/dL regardless of history of infections; shared decision making on immunoglobulin replacement shall be made for those with serum IgG < 700 mg/dL plus reduced IgA/IgM and impaired vaccine response, or serum IgG < 400 mg/dL, but without history of severe/unusual/recurrent infections [32]. Trial of antibiotic prophylaxis without immunoglobulin replacement could be given to those with history of severe/unusual/recurrent infections but without the combination of serum IgG < 700 mg/dL, reduced IgA/IgM and impaired vaccine response [32]. Otani et al. have proposed a similar framework, highlighting the growing recognition of the need for structured guidelines in this area [61]. However, their efficacy in trials and effectiveness in real-word clinical settings are yet to be validated, which represents an area for further research.

Immunoglobulin replacement should be administered at regular intervals [32, 62, 63]. A variety of regimens and dosages have been proposed and recommended, generally at a dose of around 0.4 to 0.6 g/kg/month, fine-tuned according to the clinical context [32, 59, 61]. However, a number of disease-specific guidelines provide ambiguous recommendations for SAD. For example, the iwCLL guideline only mentioned that ‘intravenous immunoglobulin should be reserved to individual situations of hypogammaglobulinemia and repeated infections’ [43]. The ESMO guideline on follicular lymphoma only mentioned ‘adequate prophylaxis (antibiotics and/or IgG supplementation) in patients with symptomatic recurrent infections and based on prior treatment (e.g. with fludarabine or bendamustine)’ [64]. Both did not specify the regimen and dosing. The European Hematology Association (EHA)-ESMO guideline on multiple myeloma and ESMO guideline on diffuse large B-cell lymphoma did not mention the management of hypogammaglobulinaemia at all [65]. In daily clinical practice, it is not uncommon for non-immunologists to give ‘as needed’ IVIg doses, i.e. giving one dose of IVIg when the patient’s IgG level falls below the threshold, and only giving the next dose when the IgG level falls below the threshold again, in lieu of regular monthly replacement [66–68]. Nevertheless, ‘as needed’ dosing of immunoglobulin administration is ineffective in preventing infections and reducing mortality among SAD patients, and should be discouraged [66–68]. 

Immunoglobulin stewardship is an increasingly discussed issue, especially given immunoglobulin’s high cost and limited availability (as a product from blood donation), as well as the rising demand in recent years [69]. Treatment of SAD had been largely based on data from primary antibody deficiencies, which are inborn errors of immunity with largely irreversible immunodeficiency, and hence there had been few data on de-escalation or cessation of treatment [8, 9]. However, SAD may be transient and hence reversible, and thus appropriate treatment de-escalation or cessation would be particularly relevant, as a contributor to immunoglobulin stewardship. Ideally, SAD patients on immunoglobulin replacement should be regularly monitored for their clinical progress, infection history, as well as immunological parameters including the trend of serum immunoglobulins and B-cell counts (with vaccine response as needed) to assess any signs of recovery, as well as consideration of de-escalation or cessation of treatment [58, 70]. Though, evidence is limited, and clinical practice has been largely dependent on clinicians’ experience and judgement, hence with great variability [71]. Multi-centre trials, such as the RATIONAL trial and RATIONALISE trial, are being carried out to explore the role of antibiotic prophylaxis relative to immunoglobulin replacement in SAD, including the possibility of cessation of immunoglobulin replacement or stepping down to antibiotic prophylaxis when immunoglobulin recipients have been free from infection for some time [72, 73]. Data from studies in this area are much needed to inform the appropriate conditions to de-escalate from immunoglobulin replacement among SAD patients, in order to optimise patient management and the practice of immunoglobulin stewardship.

### Vaccination in the Diagnosis and Management of SAD

Specific antibody response to vaccinations is a useful diagnostic tool for SAD. It can help establish the diagnosis of SAD in suspected patients with normal immunoglobulin levels, as well as determine the severity and guide treatment decisions including the use of immunoglobulin replacement in patients with hypogammaglobulinaemia [32]. Pneumococcal polysaccharide vaccine, tetanus toxoid vaccine and diphtheria toxoid vaccine are the most commonly used. Specific antibody titres are measured before and 4–8 weeks after vaccination [74]. Regarding deficient specific antibody responses to pneumococcal polysaccharide vaccine, a ‘mild’ phenotype is defined as failure to generate protective titre (1.3 µg/mL) or failure to achieve 2-fold increase in at least 70% (above 5 years of age) or 50% (5 years of age or below) of serotypes; a ‘moderate’ phenotype is defined as failure to generate protective titre (1.3 µg/mL) in at least 70% (above 5 years of age) or 50% (5 years of age or below) of serotypes (but being able to generate protective titre in more than 2 serotypes); a ‘severe’ phenotype is defined as failure to achieve protective titre (1.3 µg/mL) in more than 2 serotypes; memory phenotype is defined as loss of the vaccine response within 6–12 months after vaccination [74]. For tetanus toxoid vaccine, an impaired vaccine response is defined as failure to achieve both protective titre (0.1–0.2 IU/mL) and 4-fold increase in titre [75]. For diphtheria toxoid vaccine, an impaired vaccine response is defined as failure to achieve both protective titre (0.01–0.1 IU/mL) and 4-fold increase in titre [75]. 

Vaccinations are important in preventing infections in the context of SAD. Vaccinations are best given before immunosuppressive treatment, ideally at least 4 weeks prior to allow sufficient time for adaptive immune response to be mounted [76]. During treatment, immune response to vaccines could be diminished. It has been recommended that for B-cell depleting therapy recipients, vaccinations shall be given at least 6 months after the last dose of treatment [76]. For patients with persistent SAD that fails to recover, although humoral response to vaccines is expected to be reduced, vaccination could still be beneficial, as T-cell response may be mounted. For example, mRNA COVID-19 vaccine induced robust T-cell responses in anti-CD19 CAR-T recipients with B-cell aplasia [77]. Therefore, vaccinations as per national guidance for immunocompromised patients are recommended for patients with ongoing SAD [32]. 

## Emerging Strategies and Therapeutics in Managing SAD

**Fig. 1:**
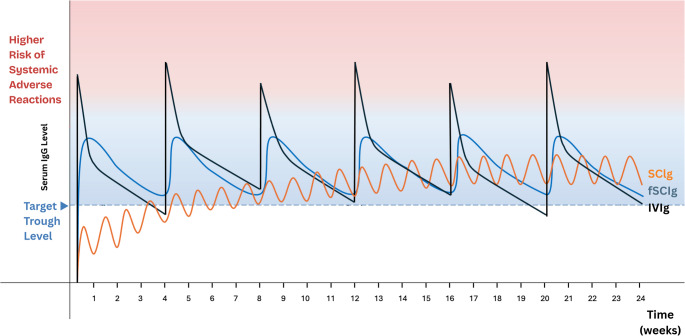
Comparison of the changes in serum IgG levels along the course of IVIg, SCIg and fSCIg replacement

Immunoglobulin replacement utilises normal human immunoglobulin, which is a pooled plasma-derived product from blood donation, primarily consisting of IgG [78]. Traditional IVIg requires monthly intravenous infusion with a large dose, raising the serum IgG to a high level, and allowing it to gradually drop over a month to the trough level as immunoglobulin is removed from the circulation by physiological mechanisms, when the next dose of IVIg is given (Fig. 1) [79]. Since the trough IgG level has to be maintained above the minimum protective level (e.g. ~600–800 mg/dL) to confer adequate protection, the IVIg dose has to be large enough, and thus the serum IgG immediately after IVIg infusion (i.e. peak level) could reach above ~ 1,800 to 2,000 mg/dL [80–82]. However, this high serum IgG level could lead to various adverse effects, including headache, myalgia, back pain, arthralgia, fever, chills and flushing, which can occur in more than 40% of patients [83]. This also predisposes to potential thromboembolic complications, such as deep vein thrombosis and stroke [84, 85]. Though these adverse effects were more reported in earlier studies, when IVIg products were carbohydrate-stabilised and older purification methods were employed. Elevated levels of activated factor XI and of factor XII were important risk factors for thromboembolic events with IVIg, and modern IVIg products have much lower levels of these pro-coagulants [86]. Apart from that, significant serum IgG trough level and peak-to-trough variations in the monthly cycles often result in a ‘wear-off effect’, where the trough level falls below the protective threshold in the third to fourth week, increasing the risk of breakthrough infections [87, 88]. Moreover, IVIg requires intravenous access; although home IVIg infusion is possible with nurse outreach support in some countries like the United States, in many countries without such service, the infusion has to be carried out in a day-hospital or clinic setting by healthcare professionals, thus requiring the patient to frequently attend healthcare facilities for their essential infusions. This can significantly disrupt daily life, employment and overall quality of life [63, 89].

SCIg has emerged as a promising alternative route to intravenous administration. Their differences are summarised in Table 3 and Fig. 1. In contrast to IVIg, a smaller dose is infused subcutaneously usually once per week (though the frequency can range from daily to bi-weekly). In terms of pharmacokinetics, unlike IVIg which the immunoglobulin immediately and directly enters the systemic circulation, in SCIg the immunoglobulin first accumulates in the subcutaneous tissues, and is slowly absorbed into the systemic circulation over a period of time [90]. While the peak IgG level is achieved within 15 min of completion of IVIg infusion, IgG level peaks around 2 to 3 days after SCIg. The more frequent dosing and the subcutaneous nature of administration in SCIg results in more stable serum IgG levels [91]. The peak-trough serum IgG difference could reach 900 mg/dL in IVIg, and the difference is less than 100 mg/dL in SCIg [92]. This reduces the systemic side effects and improves patient satisfaction [83, 93]. Also, the more consistent serum IgG levels in SCIg eliminate the wear-off effect at the end of infusion cycle and thus the risk of breakthrough infections [94, 95]. Besides, in the context of protein-losing states, traditionally most practitioners tend to be more conservative in using immunoglobulin replacement due to the concern of rapid antibody loss following the rapid surge with IVIg replacement [59]. SCIg replacement, with more stable serum IgG levels, could possibly increase the utility of immunoglobulin replacement in SAD due to protein loss [32, 96].

**Table 3:** Differences between IVIg, SCIg and fSCIg

	IVIg	SCIg	fSCIg
Infusion site	Superficial veins (venous access required).	Subcutaneous tissues, e.g. abdomen, thighs, outer buttocks (venous access not required).	Subcutaneous tissues, e.g. abdomen, thighs, outer buttocks (venous access not required).
Timing and setting	Given once every 3–4 weeks regularly, each infusion lasting for 2–4 h. Requires administration by a healthcare professional (e.g. nurse), hence mostly done in healthcare facilities (e.g. day-hospitals, clinics or infusion centres). Home infusion is possible with nurse outreach support in some countries.	Given once every 1–2 weeks regularly, each infusion lasting for 1–1.5 h. Usually self-administered.	Given once every 3–4 weeks regularly, each infusion lasting for 2–3 h. Can be administered by a healthcare professional or self-administered.
IgG levels after infusion	Serum IgG level is immediately raised to a hyper-physiological level after infusion, then reduced gradually with time. The next infusion is performed after 3–4 weeks, when the serum IgG level approaches the minimum target. Hence, there are greater variations in the serum IgG level throughout the cycle.	With the slower rate of absorption and more frequent dosing regimen, a steady physiological level of serum IgG can be maintained throughout the cycle.	With the slower rate of absorption, there is no sharp peak of serum IgG level immediately after infusion. Physiological level of serum IgG with narrower peak-to-trough variation than IVIg can be maintained throughout the cycle.
Effectiveness	Effective in protection against infections. Can rapidly confer protection in patients with severe hypogammaglobulinaemia.	Effective in protection against infections. In patients with severe hypogammaglobulinaemia, it would require several cycles to reach adequate serum IgG level to confer protection against infection if started on SCIg de novo; daily loading doses could be given during the initiation of SCIg replacement.	Effective in protection against infections (largely based on data in primary antibody deficiency; limited data in SAD)
Adverse events related to immunoglobulin replacement	More systemic adverse effects (e.g. headache, dizziness/nausea, fatigue/lethargy, dyspnoea)	Fewer systemic adverse effects. More mild local infusion site reactions.	Fewer systemic adverse effects than IVIg, similar to SCIg More local infusion site reactions than IVIg and SCIg
Quality of life	Relatively poorer HRQoL.	Relatively better HRQoL.	Relatively better HRQoL

*fSCIg* facilitated subcutaneous immunoglobulin, *HRQoL* health-related quality of life, *IVIg* intravenous immunoglobulin, *SAD* secondary antibody deficiency, *SCIg* subcutaneous immunoglobulin

Earlier studies and protocols suggested a 1:1.37 (16% IgG) or 1:1.53 (20% IgG) conversion ratio for IVIg to SCIg [97]. Later, the U.S. Food and Drug Administration recommended the conversion factor of 1:1.37 for all SCIg products (20% IgG) as well [98]. However, recent studies suggest that 1:1 dose conversion could be adequate, and at the same dose, SCIg can lead to higher trough/steady-state IgG levels than IVIg [99–101]. In a real-world cohort study on adult antibody deficiency patients, patients on SCIg required lower doses of immunoglobulin per kg of body weight compared to IVIg while achieving similar serum IgG trough/steady-state levels and infection rates [1]. Another study on patients switching from IVIg to SCIg also found that they could maintain similar trough/steady-state IgG levels after switching to a lower dose of SCIg [101]. Although much of the existing data pertain to primary antibody deficiency, the optimal dosing strategy in SAD remains an area requiring further investigation. Furthermore, the majority of patients have no difficulty with self-administration of SCIg after training, even in elderly patients [102]. Using an infusion pump, patients can do the infusion over 1–2 h at any time of the day at their convenience. This minimises the negative impact on daily life and work, and greatly enhances patients’ health-related quality of life (HRQoL) [103, 104]. 

Facilitated subcutaneous immunoglobulin (fSCIg) is a variant of the conventional SCIg, and it is another emerging modality of immunoglobulin replacement (Table 3). The infusion of recombinant human hyaluronidase prior to immunoglobulin infusion allows temporary break-down of hyaluronan in the extracellular matrix of subcutaneous tissue, enabling accommodation of a larger volume of immunoglobulin to be administered in a single infusion [105]. This allows fSCIg to be infused monthly [106]. Same as conventional SCIg, fSCIg can be self-administered at home [90]. With the slow absorption of immunoglobulin from the subcutaneous tissue to the systemic circulation, there is no sharp hyper-physiological peak of serum IgG level immediately after infusion, and there is smaller peak-to-trough variation (Fig. 1) [106]. Hence, it is associated with fewer systemic adverse effects than IVIg [107]. fSCIg was associated with better HRQoL than IVIg, and similar to conventional SCIg [108]. In short, fSCIg combines the advantages of IVIg in terms of less frequent infusions, with the advantages of SCIg in terms of steadier serum IgG level and avoidance of sharp hyper-physiological peak, fewer systemic adverse reactions, possibility of self-administration, and better HRQoL. However, since a larger volume of immunoglobulin is administered in each infusion, fSCIg is associated with more local infusion site reactions than conventional SCIG replacement [109]. The pharmacokinetics, efficacy and safety profile of fSCIg were largely based on studies in primary antibody deficiency. There have so far been few studies on fSCIg among SAD patients [110, 111]. More comparative studies are needed to establish the effectiveness, adverse reaction profile and HRQoL of fSCIg replacement relative to IVIg and SCIg in SAD.

## Comparative Effectiveness of Different Modes of Immunoglobulin Replacement in SAD

A literature review identified a total of 15 clinical studies on the use of SCIg among SAD patients, and they are listed in Table 4 [1, 93, 112–124].

**Table 4:** Summary of clinical studies on SCIg among secondary antibody deficiency patients

Studies	Patient Characteristics	IVIg vs SCIg	Treatment	(Trough) serum IgG level (mg/dL)	Infection outcomes	Adverse effects	HRQoL
*All causes of secondary antibody deficiency*							
Subcutaneous immunoglobulins replacement therapy in secondary antibody deficiencies: Real life evidence as compared to primary antibody deficiencies[112]	Secondary antibody deficiency of any cause (*N* = 131)	No	SCIg, mean dose 0.25 g/kg/month	Baseline: 368 ± 116 After at least 12 weeks of SCIg: 680 ± 171	Infection rate: 1.01 ± 1.38 (before treatment) → 0.24 ± 0.44 (at least 12 months after SCIg) episodes per patient-year	Local skin rash: 7.6% Infusion site pain: 2.3% Headache: 2.3% Mild nausea: 2.3% Chills: 1.5% Delayed fever: 1.5% Skin infection at infusion site: 0.8%	N/A
Home-based subcutaneous immunoglobulin G replacement therapy under real-life conditions in children and adults with antibody deficiency[113]	Primary antibody deficiency (*N* = 75) Secondary antibody deficiency (*N* = 9)	Yes (Sequential)	SCIg, mean dose 0.37 g/kg/month (20 patients were on IVIg of a mean dose of 0.39 g/kg/month before the start of study)	During IVIg: 660 (mean) During SCIg: 750 (mean)	N/A	Local tissue reaction: 2.4%	Statistically significant improvement in ‘Bodily Pain’, General Health’, and ‘Vitality’ domains of SF-36 at 290 days after SCIg treatment
Immunoglobulin replacement therapy in patients with immunodeficiencies: impact of infusion method on patient-reported outcomes[114]	Primary antibody deficiency (*N* = 239) Secondary antibody deficiency (*N* = 57)	Yes (Head-to-head)	IVIg (*N* = 54) vs SCIg (*N* = 242)	N/A	Antibiotic use since the initiation of treatment: 78.6% (IVIg) vs 60.4% (SCIg)	N/A	Significantly better HRQoL in TSQM-9 Effectiveness, and Patient Acceptable Symptom State (PASS) among those on SCIg
Clinical outcomes of immunoglobulin treatment for patients with secondary antibody deficiency: Data from the Ontario immunoglobulin treatment case registry[115]	Secondary antibody deficiency of any cause (*N* = 140)	No	IVIg, median dose 0.48 g/kg/month (*N* = 9), SCIg, median dose 0.44 g/kg/month (*N* = 131)	Baseline: 370 (235–485) After immunoglobulin replacement: 800 (645–960)	Infection rate: 4.6 (before treatment) → 0.8 (after immunoglobulin replacement) episodes per patient-year	N/A	Subjective improvement in health in 84.6%
Initiation of immunoglobulin therapy by subcutaneous administration in immunodeficiency patients naive to replacement therapy[116]	Primary antibody deficiency (*N* = 11) Secondary antibody deficiency (*N* = 2)	No	SCIg, 0.1 g/kg/week	Baseline: 460 ± 146 After 1 month of SCIg: 852 ± 106 After 3 months of SCIg: 907 ± 157 After 6 months of SCIg: 943 ± 182	1 episode of serious bacterial infection (sepsis/cholangitis) was recorded among the 13 patients, with a median duration of treatment and follow-up of 44 months	Systemic adverse reactions: 0% Mild local infusion site reactions: 46% Moderate local infusion site reactions: 15%	N/A
Ten-year population trends of immunoglobulin use, burden of adult antibody deficiency and feasibility of subcutaneous immunoglobulin (SCIg) replacement in Hong Kong Chinese[1]	Primary antibody deficiency (*N* = 16) Secondary antibody deficiency (*N* = 6)	Yes (Head-to-head)	IVIg, mean dose 0.66 g/kg/month (*N* = 14) vs SCIg, mean dose 0.52 g/kg/month (*N* = 8)	Baseline: 309 ± 264 (IVIg) vs 490 ± 251 (SCIg) During immunoglobulin replacement: 1086 ± 236 (IVIg) vs 1073 ± 135 (SCIg)	Infection rate: 4.86 ± 4.37 (IVIg) vs 3.13 ± 3.09 (SCIg)	Fever: 28.6% (IVIg) vs 0% (SCIg) Fatigue: 28.6% (IVIg) vs 0% (SCIg) Sickness/dizziness/headache/nausea: 21.4% (IVIg) vs 0% (SCIg) Dyspnoea: 7.1% (IVIg) vs 0% (SCIg) Infusion site reactions: 64.3% (IVIg) vs 75.0% (SCIg)	Better HRQoL in SF-36v2 and Life Quality Index (LQI) among those on SCIg
*Haematological malignancies*							
Subcutaneous immunoglobulin in lymphoproliferative disorders and rituximab-related secondary hypogammaglobulinemia: a single-center experience in 61 patients[117]	Lymphoproliferative disorders with secondary antibody deficiency (*N* = 61, with 49 of them treated with rituximab)	Yes (Sequential)	SCIg, mean dose 0.075 g/kg/week (33 patients had previous IVIg, mean dose 0.32 g/kg/month)	Baseline: 380 ± 119 After IVIg: 474 ± 116 After SCIg: 660 ± 173	Infection rate: 2.79 (before treatment) → 2.29 (during IVIg) → 1.76 (during SCIg) episodes per patient-year Antibiotic use: 2.35 (before treatment) → 1.82 (during IVIg) → 1.43 (during SCIg) cycles per patient-year	Infusion site reactions: 0% in IVIg, 10% in SCIg Fever: 33% in IVIg, 7% in SCIg Sickness/dizziness/headache/nausea: 9% in IVIg, 2% in SCIg Diffuse skin reactions: 15% in IVIg, 0% in SCIg Dyspnoea: 9% in IVIg, 0% in SCIg Anaphylaxis: 3% in IVIg, 0% in SCIg	Generally subjective improvement in quality of life after switching from IVIg to SCIg
Subcutaneous immunoglobulins in chronic lymphocytic leukemia with secondary antibody deficiency. A monocentric experience during Covid-19 pandemics[118]	Chronic lymphocytic leukaemia with secondary antibody deficiency (*N* = 10)	No	SCIg	Baseline: 485 (118–817) 6 months after SCIg: 615 (436–865) 12 months after SCIg: 602 (538–915)	No infections during the one-year study period during the COVID-19 pandemic	One patient (10%) developed a grade 2 skin rash (Common Terminology Criteria for Adverse Events 4.0)	All reported subjective improvement in quality of life
A Retrospective Study on the Efficacy of Subcutaneous Immunoglobulin as Compared to Intravenous Formulation in Patients with Chronic Lymphocytic Leukemia and Secondary Antibody Deficiency[93]	Chronic lymphocytic leukaemia with secondary antibody deficiency (*N* = 116)	Yes (Head-to-head)	IVIg (*N* = 49) vs SCIg (*N* = 88) (21 patients switched from IVIg to SCIg, and hence counted in both categories in the study)	Baseline: 380 ± 120 (IVIg) vs 390 ± 100 (SCIg) 6 months after treatment: 520 ± 120 (IVIg) vs 600 ± 140 (SCIg) 12 months after treatment: 520 ± 180 (IVIg) vs 620 ± 150 (SCIg)	Infection rate: IVIg: 2.31 (before treatment) → 3.14 (during treatment) episodes per patient-year SCIg: 2.59 (before treatment) → 1.43 (during treatment) episodes per patient-year 4-year cumulative incidence of first infection: 91% (IVIg) vs 58% (SCIg)	Any adverse reactions: 20.8% (IVIg) vs 5.6% (SCIg) Fever: 16.3% (IVIg) vs 2.3% (SCIg) Skin rash: 7.2% (IVIg) vs 3.2% (SCIg) Headache/dizziness/nausea: 8.2% (IVIg) vs 0% (SCIG) Dyspnoea: 6.1% (IVIg) vs 0% (SCIg)	N/A
Subcutaneous gammaglobulin for patients with secondary hypogammaglobulinaemia[119]	Chronic lymphocytic leukaemia (*N* = 14), Waldenstrom’s disease (*N* = 2), lymphoma (*N* = 1) with secondary antibody deficiency	No	SCIg, 0.05 g/kg/week	Baseline: 310 (mean) 6 months after SCIg: 550 (mean)	Infection rate: 0.63 (before treatment) → 0.42 (during treatment) episodes per patient-year Antibiotic use: 1.74 (before treatment) → 1.45 (during treatment) courses per patient-year	N/A	N/A
Longitudinal study of intravenous versus subcutaneous immunoglobulin replacement therapy in hematological malignancy[120]	Haematological malignancy with secondary antibody deficiency (*N* = 17, with 14 of them treated with rituximab, and 6 of them treated with HSCT, respectively)	Yes (Sequential)	SCIg (13 patients were on IVIg before the start of study)	During IVIg: 700 ± 277 During SCIg: 800 ± 175 (first year), 870 ± 275 (second year), 760 ± 289 (third year)	Infection rate: 2.08 ± 2.14 (during IVIg) → 2.06 ± 1.52 (first year of SCIg) → 1.58 ± 1.54 (second year of SCIg) -> 1.65 ± 2.23 (third year of SCIg) episodes per patient-year	Lethargy: 66.7% (IVIg) vs 40% (SCIg) Headache: 44.4% (IVIg) vs 20% (SCIg) Muscle pain: 33.3% (IVIg) vs 33.3% (SCIg) Vomiting/nausea: 22.2% (IVIg) vs 6.67% (SCIg) Skin rash/itchiness: 11.1% (IVIg) vs 20% (SCIg)	Improved quality of life among 75% who switched from IVIg to SCIg
*Transplantation*							
Subcutaneous immunoglobulin in allogeneic hematopoietic cell transplant patients: A prospective study of feasibility, safety, and healthcare resource use[121]	Post-allogeneic HSCT with secondary antibody deficiency (*N* = 40)	Yes (Head-to-head)	IVIg, 0.4 g/kg/28 days (*N* = 20) vs SCIg, 0.1 g/kg/week for up to 6 months (*N* = 20)	N/A	Infections: 10 episodes in total in 6 months (IVIg) vs 24 episodes in total in 6 months (SCIg)	Infusion site reactions: 0% (IVIg) vs 30% (SCIg)	Improved EQ-5D score compared to baseline in the SCIg group
Subcutaneous IgG replacement therapy is safe and well tolerated in lung transplant recipients[122]	Post-lung transplantation with secondary antibody deficiency (*N* = 10)	No	SCIg, 0.1 g/kg/week	Baseline: 422 (383–626) 3 months after SCIg: 1,065 (724-1,259) 6–12 months after SCIg: 892 (805-1,195)	N/A	Infusion site reactions: 30%	N/A
*Iatrogenic*							
Immunoglobulin G replacement for the treatment of infective complications of rituximab-associated hypogammaglobulinemia in autoimmune disease: a case series[123]	Post-rituximab treatment for systemic autoimmune disease with secondary antibody deficiency (*N* = 12)	Yes (Sequential)	IVIg starting dose at 0.4 g/kg/month, subsequently titrated to achieve serum trough IgG 800-1,000 mg/dL, then switching to SCIg after stabilisation	Lowest level before immunoglobulin replacement: 320 (238–383) After IVIg/SCIg: N/A	Generally decreased frequency and severity of infections	N/A	N/A
Intravenous versus subcutaneous immunoglobulin replacement in secondary hypogammaglobulinemia[124]	Post-rituximab treatment for non-Hodgkin lymphoma (*N* = 12) and chronic lymphocytic leukaemia (*N* = 2) with secondary antibody deficiency	Yes (Sequential)	IVIg (mean dose 0.4 g/kg/month), then switching to SCIg (mean dose 0.1 g/kg/week)	Baseline: 251 ± 106 During IVIg: 599 ± 116 During SCIg: 846 ± 168	Infection rate: 11.1 ± 3.0 (baseline) → 3.0 ± 1.4 (during IVIg) → 2.4 ± 1.4 (during SCIg) episodes per patient-year	N/A	N/A

*HRQoL* health-related quality of life, *IVIg* intravenous immunoglobulin, *N/A* not available/not applicable, *SCIg* subcutaneous immunoglobulin, *SF-36* SF-36 Health Survey, *TSQM* Treatment Satisfaction Questionnaire for Medication

### Trough/Steady-State Serum IgG Levels

The mean/median trough/steady-state serum IgG levels after SCIg in most studies were above 600 mg/dL [1, 93, 112, 113, 115–118, 120, 122, 124], except for one which only raised the mean trough level to 550 mg/dL, which was most likely due to the relatively low dose of SCIg used in that study (200 mg/kg/month) [119]. Sequential studies found higher trough/steady-state serum IgG levels after switching from IVIg to SCIg, despite lower mean doses of immunoglobulin being used [113, 117, 124]. Another study also found higher trough/steady-state levels after switching, but the doses of immunoglobulin used per body weight were not specified [120]. A head-to-head comparative study demonstrated significantly higher trough/steady-state serum IgG levels among patients on SCIg than those on IVIg, despite similar baseline serum IgG levels [93]. Another head-to-head comparative study showed similar baseline and trough/steady-state serum IgG levels among those on SCIg and IVIg, but the doses of immunoglobulin required were significantly lower among those on SCIg [1]. These findings suggest that SCIg may achieve higher trough/steady-state serum IgG levels than IVIg at similar doses among patients with SAD.

### Infection Outcomes

Infection rates significantly reduced after SCIg. Longitudinal studies found reduced infection rates after SCIg compared to baseline [112, 115, 119]. Subsequent sequential studies revealed significantly reduced infection rates after switching from IVIg to SCIg, from 2.08–3.0 episodes per patient-year while on IVIg to 1.65–2.4 episodes per patient-year while on SCIg [117, 120, 124]. Head-to-head comparative studies also showed lower infection rates among those on SCIg (1.43–3.13 episodes per patient-year) compared to those on IVIg (3.14–4.86 episodes per patient-year) [1, 93]. However, among allogeneic HSCT recipients with SAD, Pasic et al. recorded more infections among the SCIg group than the IVIg group within a 6-month period, though 30% of the SCIg recipients were naïve to immunoglobulin replacement, and the dose used in both arms was the same (0.4 g/kg/month) in this study [121]. 

There was a reduction in antibiotic courses, from 1.74 per patient-year at baseline to 1.45 per patient-year during SCIg [119]. In a sequential study, antibiotic consumption reduced from 2.35 cycles per patient-year at baseline, to 1.82 cycles per patient-year during IVIg, and finally 1.43 cycles per patient-year during SCIg [117]. A head-to-head comparison study revealed that 78.6% of those on IVIg used antibiotics since the initiation of immunoglobulin replacement compared to 60.4% among those on SCIg [114]. 

These findings demonstrate that SCIg is efficacious in reducing infections, and may be relatively more effective than IVIg in SAD. However, in severe cases of hypogammaglobulinaemia, initiating treatment with SCIg directly may not be appropriate, as it would take a longer period of time to reach stable and protective levels of serum IgG. One study using pharmacokinetic model simulations showed that starting a naïve patient with severe hypogammaglobulinaemia on SCIg might take up to 24 weeks to reach steady-state serum IgG levels [125]. In such situations, patients could be started with IVIg in order to confer immediate protection, and after stabilisation, they could be switched to SCIg. For example, package inserts of SCIg products recommended a naïve patient initiating SCIg replacement to receive one IVIg infusion, then start with SCIg one week after the IVIg infusion [126]. Alternatively, SCIg may be administered in daily loading doses during the initiation of SCIg replacement (e.g. the first 5 days) to more rapidly achieve protective serum IgG levels [127]. 

### Adverse Effects

SCIg was associated with fewer systemic adverse effects than IVIg in SAD. While 16% to 33% of those on IVIg suffered from fever, only 0% to 7% of those on SCIg had experienced fever as an adverse reaction [1, 93, 112, 117, 120]. Headache was reported by 8% to 44% of patients on IVIg, and 0% to 20% of those on SCIg [1, 93, 112, 117, 120]. Sickness/dizziness/nausea were reported by 8% to 22% of patients on IVIg, and only 0% to 7% of those on SCIg [1, 93, 112, 117, 120]. Fatigue/lethargy was experienced by 29% to 67% of those on IVIg, and 0% to 40% of those on SCIg [1, 120]. While 6% to 9% of patients on IVIg reported dyspnoea, none (0%) of the patients on SCIg had dyspnoea [1, 93, 117]. 

However, SCIg was generally associated with more local infusion site reactions than IVIg. One study reported local skin rash in 7.6%, infusion site pain in 2.3%, and skin infection at the infusion site in 0.8% of the patients on SCIg [112]. Another study found that despite none of the patients on SCIg suffered from systemic adverse reactions, mild and moderate local infusion site reactions occurred in up to 46% and 15% of the patients respectively [116]. One other study also found that 30% of patients on SCIg experienced infusion site reactions [122]. All 2 sequential studies and 2 of the 3 head-to-head studies with comparative data on adverse effects available showed more infusion site reactions among those on SCIg (ranging from 10 to 75%) than those on IVIg (ranging from 0 to 64.3%) [1, 117, 120, 121]. Only one head-to-head study reported more skin rash among those on IVIg than those on SCIg (7.2% vs 3.2%) [93]. Nevertheless, according to phase III clinical study data, local infusion site reactions with SCIg were only relatively more prominent in the initial phase of treatment, and they decreased over time in subsequent infusions [99, 128]. 

Though, the capture of adverse events in these clinical studies was variable. Prospective safety data on SAD patients are warranted to validate the adverse event profile of IVIg versus SCIg replacement in this group of patients. This highlights the need for dedicated clinical trials directly comparing IVIg and SCIg replacement among SAD patients in future research.

### Health-Related Quality of Life (HRQoL)

Among the 15 studies, 8 of them evaluated patients’ HRQoL. However, only half of these studies employed validated HRQoL measures, while the other half only reported patients’ subjective quality of life [1, 93, 112–124]. There was statistically significant improvement in 3 out of 8 domains of SF-36 (‘Bodily Pain’, ‘General Health’, and ‘Vitality’) at 290 days after SCIg treatment compared to baseline (before any immunoglobulin replacement) [113]. EQ-5D scores also improved after SCIg compared to baseline [121]. A head-to-head comparative study revealed significantly better HRQoL in Treatment Satisfaction Questionnaire for Medication (TSQM)-9 Effectiveness, and Patient Acceptable Symptom State (PASS) [114], while another head-to-head study also demonstrated better HRQoL in SF-36v2 and Life Quality Index (LQI) among those on SCIg compared to those on IVIg [1]. Other studies also found subjective improvements in quality of life in the majority of patients on SCIg compared to previous IVIg [115, 117, 118, 120]. 

## Cost-Effectiveness of SCIg in SAD

The cost-effectiveness of SCIg compared to IVIg has been well-established in primary antibody deficiency [129–132]. On the contrary, there were only 2 studies investigating the cost-effectiveness of SCIg in SAD patients, and another 2 studies including both primary and SAD patients.

The cumulative cost of IVIg and SCIg for 10 years could be Australian Dollars (AUD)151,511 (United States Dollars [USD]90,976) and AUD144,296 (USD86,644) respectively, and the quality-adjusted life years (QALY) using IVIg and SCIg would be 3.07 and 3.51 respectively, meaning that higher QALY can be achieved with a lower cost using SCIg [133]. In a head-to-head comparative study, the total median costs over the 6 months for IVIg and SCIg were Canadian Dollars (CAD)13,780 (USD9,696) and CAD9,756 (USD6,865) respectively, which included costs of drug and drug administration, antimicrobials, hospitalisations, clinical consultations, as well as laboratory tests [121]. 

A health economic study showed that switching primary and SAD patients from IVIg to SCIg would result in a net reduction of healthcare cost of USD1,832 per patient in the first year and USD2,074 per patient in each subsequent year [134]. In terms of manpower, one nurse full-time equivalent would be recouped by switching every 37 patients from IVIg to SCIg [134]. In a real-world head-to-head comparative study of IVIg and SCIg among adult primary and SAD patients, the healthcare costs (including costs of drugs and hospitalisations) of IVIg and SCIg per patient-year were USD28,319 and USD25,096 respectively, which means the cost could be reduced by USD3,221 per patient-year on SCIg compared to IVIg [1]. 

The above suggests that SCIg may be more cost-effective than IVIg among SAD patients. Nevertheless, the cost of immunoglobulin replacement is a complex issue that varies internationally, depending on a number of factors including negotiated national acquisitions, negotiated payer relationships, and national styles of healthcare delivery, which could affect both the drug cost and the treatment-associated healthcare costs, and hence the overall cost-effectiveness. Hence, more dedicated and region-specific health economic studies are needed to establish the cost-effectiveness of SCIg relative to IVIg replacement across different localities and contexts.

## Future Research Directions

Further research should prioritise enhanced diagnostic strategies and screening protocols. There is a clear need to establish consensus guidelines among healthcare providers, particularly among non-immunologists. Future studies should focus on developing standardised screening protocols in at-risk populations. This includes validating the effectiveness of routine screening with immunoglobulin profile among patients with underlying conditions such as haematological malignancies or those receiving immunosuppressive therapies. Further studies shall also explore the role of additional immunological tests such as specific antibodies and vaccine responses, IgG subclass, lymphocyte subset and B-cell phenotyping in screening and monitoring for SAD. Research initiatives should also address disparities in the management of SAD across different regions. Efforts to standardise education for clinicians regarding antibody deficiencies can ensure comprehensive understanding and adherence to updated clinical practices and guidelines.

For the treatment of SAD, most existing studies have a relatively small sample size. There is a critical need for large-scale multi-centre clinical trials and real-world studies directly comparing IVIg, SCIg and fSCIg among SAD patients to enable the formulation of more specific high-quality evidence-based recommendations on the choice of treatment modality across different situations. In existing real-world/non-randomised studies, newly diagnosed SAD patients with low serum IgG levels and higher infection risk may be preferentially started with IVIg, leaving room for potential bias and confounding factors. This further highlights the need for well-designed randomised controlled trials. Research into the cost-effectiveness of different immunoglobulin replacement methods is also essential, particularly in various healthcare contexts and geographies.

Future research should also consider viral infections, such as measles, rubella, Epstein-Barr virus, and human immunodeficiency virus, which are possible causes of SAD [6]. Some of which, such as measles and rubella, are vaccine-preventable. These infections can impair humoral immunity and contribute to increased susceptibility to additional infections, especially in regions where vaccine coverage is suboptimal or disrupted. Addressing these viral aetiologies is particularly relevant especially given the doubts of immunisation practices in some countries.

## Conclusion

In conclusion, this review highlights the increasingly recognised yet often overlooked burden of SAD, a condition that far exceeds the prevalence of primary antibody deficiencies yet remains under-researched and under-diagnosed. Ultimately, addressing the challenges associated with SAD is essential not only for enhancing individual patient care but also for relieving the broader global health burdens associated with untreated or inadequately managed immunodeficiencies. Greater collaboration across clinical, research, and policy frameworks will be vital to advance our understanding and management of this complex condition in the years to come.

## Data Availability

No datasets were generated or analysed during the current study.
